# Identification of a novel splicing mutation in the *SLC25A13* gene from a patient with NICCD: a case report

**DOI:** 10.1186/s12887-019-1751-9

**Published:** 2019-10-13

**Authors:** Linlin Zhang, Yingying Li, Wenli Shi, Jinshuang Gao, Yuan Tian, Ying Li, Yaqing Guo, Shihong Cui, Xiaoan Zhang

**Affiliations:** 1 Department of Clinical Laboratory, The Third Affiliated Hospital of Zhengzhou University, Zhengzhou, 450052 Henan Province China; 2 International Joint Research Laboratory for US-China Prenatal Medicine Of Henan, Zhengzhou, China; 3 Department of Obstetrics and Gynecology, The Third Affiliated Hospital of Zhengzhou University, Zhengzhou, 450052 Henan Province China

**Keywords:** *SLC25A13* gene, NICCD, Splicing mutation, Amplicon sequencing, Pedigree analysis

## Abstract

**Background:**

Neonatal intrahepatic cholestasis caused by citrin deficiency (NICCD) is an autosomal recessive disorder and one of the most common inherent causes of cholestatic jaundice in Asian infants. Mutations in the *SLC25A13* gene, which encodes citrin protein expressed in the liver, have been identified as the genetic cause for NICCD.

**Case presentation:**

Here, we report a 4-month-old female with clinical features including jaundice, hyperbilirubinemia, hyperlactacidemia, and abnormal liver function. The patient was diagnosed with NICCD by differential diagnosis using genetic analysis. Mutations in 60 jaundice-related genes were tested by using amplicon sequencing, which was performed on an Ion S5XL genetic analyzer. A compound heterozygous mutation in the *SLC25A13* gene was identified, consisting of a known deletion *SLC25A13:*c.852_855delTATG and a novel splicing mutation *SLC25A13:*c.1841 + 3_1841 + 4delAA. Sanger sequencing for the proband and her parents was performed to validate the result and reveal the source of mutations.

**Conclusion:**

A compound heterozygous mutation in the *SLC25A13* gene was identified in a 4-month-old female patient with NICCD. Our data suggest that amplicon sequencing is a helpful tool for the differential diagnosis of inherited diseases with similar symptoms. Further studies of the mutation spectrum of neonatal jaundice in China are warranted.

## Background

Citrin deficiency is an autosomal recessive disorder caused by mutations of the *SLC25A13* gene on chromosome 7q21.3. The *SLC25A13* gene is mainly expressed in the liver and encodes an aspartate glutamate carrier (AGC), which plays an important role in malate–aspartate NADH shuttling and urea synthesis [1]. There are two major clinical phenotypes of citrin deficiency, adult-onset type II citrullinemia (CTLN2, OMIM: 603471) and neonatal intrahepatic cholestatic hepatitis (NICCD, OMIM: 605814) [2, 3]. Patients with CTLN2 suffer from recurring neuropsychiatric symptoms, such as disorientation, delirium, and delusion, which are generally associated with hyperammonemia. Symptoms of NICCD patients include cholestatic jaundice, hypoproteinemia, hypoglycemia, liver enlargement and dysfunction, most of which usually disappear by 12 months of age without special treatment [4, 5]. However, some patients might develop CTLN2 decades later [6]. In addition, another phenotype different from NICCD and CTLN2 was reported in older child patients, which was referred to as failure to thrive and dyslipidemia caused by citrin deficiency (FTTDCD) [7].

In China, the frequency of carriers with mutations in the *SLC25A13* gene is very high and presents significant geographic differences. The frequency was estimated to be 1/940 and 1/48 in northern and southern China, respectively [8]. Recently, the mutation spectrum of the *SLC25A13* gene was investigated, resulting in the variations c.851_854del4, c.1638_1660dup, IVS6 + 5G > A and IVS16ins3Kb constituting the high-frequency mutations on top of the list [9]. Molecular diagnosis provides essential evidence for diagnosing citrin deficiency.

In this case, we report a 4-month-old female patient diagnosed with NICCD using amplicon sequencing of whole exons of 60 cholestatic jaundice-related genes. A novel splicing mutation, *SLC25A13:*c.1841 + 3_1841 + 4delAA, was identified in this patient, compound with a known deletion c.852_855delTATG. The two mutations were confirmed and proven to be separately inherited from father and mother by Sanger sequencing. Our data expand the mutation spectrum of the *SLC25A13* gene in Chinese patients with NICCD and demonstrate that amplicon sequencing is accurate and efficient for identifying mutations in patients with inherited diseases.

## Case presentation

This study was approved by the ethics committee of the Third Affiliated Hospital of Zhengzhou University. Informed consent from the parents was obtained before collecting blood samples.

A 4-month-old female patient was admitted to the Department of Pediatrics in the Third Affiliated Hospital of Zhengzhou University Hospital for mild neonatal jaundice. The chief complaint was jaundice, coughing and runny nose. Physical examination was normal, and ultrasonic examination showed a slight gallbladder wall thickening. Biliary atresia was excluded based on the normal result of magnetic resonance cholangiopancreatography (MRCP). Blood and liver function tests were performed in the clinical laboratory, and hyperbilirubinemia, hyperlactacidemia, and abnormal liver function were revealed, suggesting neonatal hepatitis in the patient (Table 1). Symptoms were significantly improved after 5 days of treatment, and the patient was discharged with recommendations for diet management and regular examination.

**Table 1:** Clinical features of the patient with jaundice

Index	Test result	Range
Blood cell counting		
WBC	15.80 × 10^9^/L	3.5–9.5 × 10^9^/L
RBC	3.63 × 10^12^/L	3.8–5.1 × 10^12^/L
PLT	359.0 × 10^9^/L	125.0–350.0 × 10^9^/L
Hb	94.0 g/L	115.0–150.0 g/L
Blood biochemistry		
GLU	3.80 mmol/L	4.1–6.5 mmol/L
LAC	5.25 mmol/L	0.5–2.2 mmol/L
ALT	65 U/L	4–33 U/L
AST	160 U/L	4–32 U/L
TBIL	156.6 μmol/L	3.4–20.5 μmol/L
DBIL	121.6 μmol/L	0–10 μmol/L
IBIL	35.0 μmol/L	1.5–16.5 μmol/L
TG	3.08 mmol/L	0.05–1.7 mmol/L
TBA	210.6 μmol/L	1.0–10 μmol/L
BUA	198.6 μmol/L	142.8–339.2 μmol/L
BA	57 μmol/L	18–72 μmol/L
PCT	2.92 ng/mL	0.02–0.05 ng/mL
AFP	47,328 ng/mL	< 25 ng/mL
Immunology		
IgA	0.13 g/L	0.82–4.53 g/L
IgG	5.50 g/L	6.6–17.5 g/L
IgM	0.66 g/L	0.46–3.04 g/L
C3	0.54 g/L	0.16–0.38 g/L
C4	0.11 g/L	0.22–0.58 g/L

*WBC* White blood cell, *RBC* Red blood cell, *PLT* Platelet, *Hb* hemoglobin, *GLU* Blood glucose, *LAC* Lactic acid, *ALT* glutamic-pyruvic transaminase, *AST* Glutamic oxalacetic transaminase, *TBIL* Total bilirubin, *DBIL* Direct bilirubin, *IBIL* Indirect bilirubin, *TG* Triglyceride, *TBA* Total bile acid, *BUA* Blood uric acid, *BA* blood ammonia, *PCT* Procalcitonin, *AFP* alpha fetoprotein

### Differential diagnosis for NICCD

The patient was diagnosed with neonatal hepatitis based on neonatal jaundice, hepatomegaly and liver dysfunction. Further differential diagnosis should consider three major pathogenesis, including infection, congenital biliary atresia and inherited metabolic diseases of the liver. CMV and TORCH test were negative, excluding the infectious hepatitis. The level of total bile acid (TBC) was out of normal range, suggesting the possibility of congenital biliary atresia. However, Kaolin stool wasn’t observed, MRCP was normal, which means biliary atresia was also excluded. The likely possible pathogenesis could be inherited metabolic diseases of the liver. In a retrospective study of liver function and islet beta cell functions for 36 patients with NICCD and 50 control individuals indicates that significantly higher of alpha-fetoprotein is one of the typical symptoms in NICCD patients, which is the result of liver dysfunction correlated with islet beta cell functions [10]. Combining the high frequency in Southern China, NICCD is highly suspected [9]. Considering the similar symptoms and complex causative mutations for inherited metabolic liver diseases, high-throughput sequencing provides an effective method of further differential diagnosis on genetic level.

A genetic test was performed to detect potential pathogenic mutations in this patient using high-throughput amplicon sequencing. In brief, genomic DNA was extracted from a 0.5 mL peripheral blood sample, and a custom-designed amplicon sequencing panel purchased from Thermo Fisher Scientific was used to amplify whole exons of 60 cholestatic jaundice-related genes (Additional file 1: Table S1). PCR products were processed and sequenced with the Ion Chef and Ion GeneStudio S5 System, and variants were detected and analyzed using VariantCaller V5.0. A filter was applied to exclude common variants with minor allele frequency (MAF) > 1% in the UCSC, dbSNP and 1000 Genomes Project databases. The clinical significance of the remaining candidate variants was predicted by PolyPhen-2 [11], SIFT [12] and HSF [13] (Additional file 2: Figure S1), the change of splicing site was predicted by NNSPLICE (http://www.fruitfly.org/seq_tools/splice.html) [14].

**Fig. 1:**
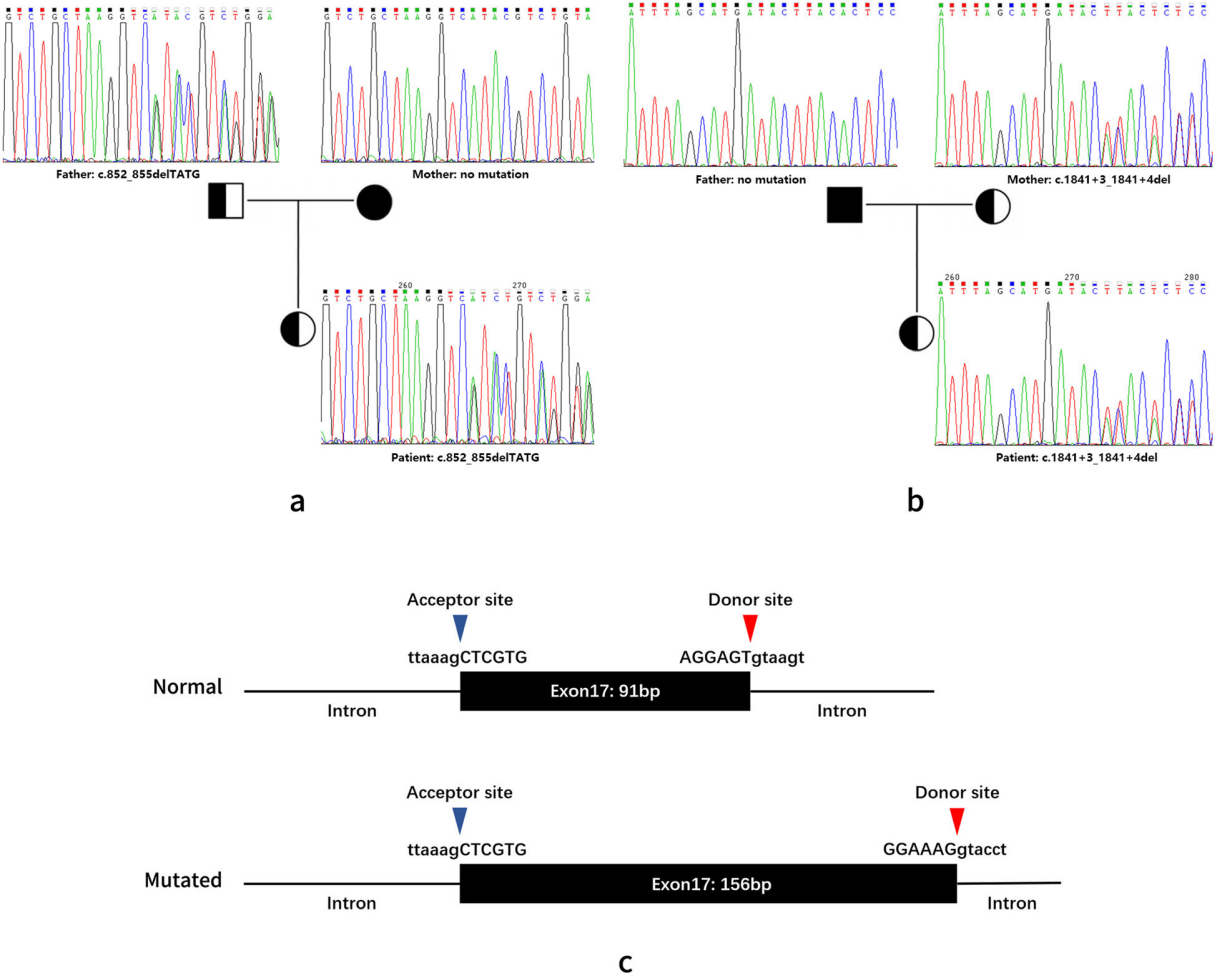
Mutation detection and splicing site prediction in the SLC*25A13* gene. **a** Identification of frame-shift deletion c.852_855delTATG using Sanger sequencing; **b** Identification of splice-site mutation c.1841 + 3_1841 + 4delAA using Sanger sequencing; **c** Change of splice-site predicted by NNSPLICE

As result, a compound heterozygote in the *SLC25A13* gene was identified and considered to be causative for this patient. The frame-shift deletion *SLC25A13:*c.852_855delTATG (NM_014251.2:c.852_855delTATG) is a pathogenic SNP (rs80338720) with a MAF = 0.0003, according to the gnomAD database. This deletion causes an amino acid sequence change in Met285Profs, which could significantly affect the function of the citrin protein. Another variant in this compound heterozygote is *SLC25A13:*c.1841 + 3_1841 + 4delAA, which is also referred to as NC_000007.13:g.95750963_95750964delTT. This variant is located in the intron between exon17 and exon18 of the *SLC25A13* gene, and it is predicted to be an alteration that most likely affects splicing. Scores predicted by both HSF Matrices and MaxEnt indicate a dramatic change caused by this mutation, probably lead to broken of the wild-type splice-site (Additional file 3: Table S2 and Additional file 4: Figure S2). A new donor site was detected 65 bp downstream, by using the NNSPLICE (Fig. 1c). This splicing mutation is reported for the first time in patient of NICCD.

To validate the result of amplicon sequencing and investigate the source of these two mutations, we analyzed these two variants for the proband and her parents using Sanger sequencing. The result shows a typical autosomal recessive inheritance pattern in this case. The c.852_855delTATG and c.1841 + 3_1841 + 4delAA mutations were inherited from the patient’s father and mother, respectively (Fig. 1a and b). Both parents are healthy, and no family history was revealed.

## Discussion and conclusions

NICCD is one of the most common inherent causes of cholestatic jaundice in Asian infants. In this case, we reported a 4-month-old female patient diagnosed with NICCD by genetic test. The patient was admitted for mild neonatal jaundice. Clinical features and results of laboratory tests suggested neonatal intrahepatic cholestasis. Considering its high frequency in south China [9], NICCD was highly suspected. However, it’s unlikely to give a diagnosis since plasma free amino acid analysis wasn’t a routine test in our clinical laboratory. To identify the pathogenic mutation in this patient, we used a custom-designed amplicon sequencing panel which aiming to make differential diagnosis for pathologic jaundice. As result, a compound heterozygote in the *SLC25A13* gene was identified in this patient, which was suggested to be causative by functional prediction. Sanger sequencing confirmed the result and revealed that two heterozygous mutations were inherited from the father and mother, which perfectly match the autosomal recessive inheritance model. The patient was discharged after 5 days of treatment, including anti-infection, liver protection, and cholagogue. Prognosis is good in regular reexamination in the following 2 months.

The frame-shift deletion *SLC25A13:*c.852_855delTATG detected in this patient is relatively rare, with MAF = 0.0003 in gnomAD, causing an amino acid sequence change in Met285Profs. It had been reported to be pathogenic in several cases in the ClinVar database. There is only one nucleotide difference between this mutation and the most frequent variation c.851_854del4; however, the prevalence of these two mutations in patients with citrin deficiency varies greatly. The mutation c.1841 + 3_1841 + 4delAA is reported for the first time, which is predicted to affect RNA splicing, resulting a new donor site downstream. Splice-site mutations are rare in the *SLC25A13* mutation spectrum, representing nearly 1/10 (4 in 41) of mutations composing the spectrum [9]. In some inherited diseases, symptoms with different severities could be caused by different genotypes [15]; however, the genotype-phenotype correlations of citrin deficiency remain unknown. A previous study had investigated these correlations initially, but no clear conclusion was established [16]. Obviously, large cohort investigations and genetic screening are required. In this case, we identified mutations within 24 h using amplicon sequencing, and the result was perfectly matched with Sanger sequencing. This indicates that amplicon sequencing is helpful for diagnosis and further studies of inherited diseases.

In summary, we identified a compound heterozygous mutation in the *SLC25A13* gene from a patient with citrin deficiency, consisting of a known deletion (c.852_855delTATG) and a novel splicing mutation (c.1841 + 3_1841 + 4delAA). Both mutations were predicted to be harmful for protein function. Pedigree analysis was performed using Sanger sequencing, demonstrating that the mutations were inherited from the patient’s father and mother. For patients with neonatal jaundice, accurate and efficient differential diagnosis is important for appropriate treatment and prognosis. Our data suggest that amplicon sequencing could play a useful role in the clinical diagnosis of inherited diseases.

## Supplementary information

**Additional file 1: Table S1.** Details of Amplicon sequencing panel for 60 cholestatic jaundice related genes.

**Additional file 2: Figure S1.** Workflow of detecting causative mutations for inherited disease.

**Additional file 3: Table S2.** Result of splice-site prediction.

**Additional file 4: Figure S2.** Result of functional prediction for SLC25A13:c.1841+3_1841+4delAA by using HSF.

## Data Availability

Not applicable.

## Abbreviations

AGC: Aspartate glutamate carrier
CTLN2: Citrin deficiency, adult-onset type II citrullinemia
MAF: Minor allele frequency
MRCP: Magnetic resonance cholangiopancreatography
NICCD: neonatal intrahepatic cholestasis caused by citrin deficiency
