# Supplementary figures and images for: Identification of a novel splicing mutation in the SLC25A13 gene from a patient with NICCD: a case report
Source: BMC Pediatr. 2019 Oct 13;19:348. doi: 10.1186/s12887-019-1751-9 (PMC6790242; doi:10.1186/s12887-019-1751-9)

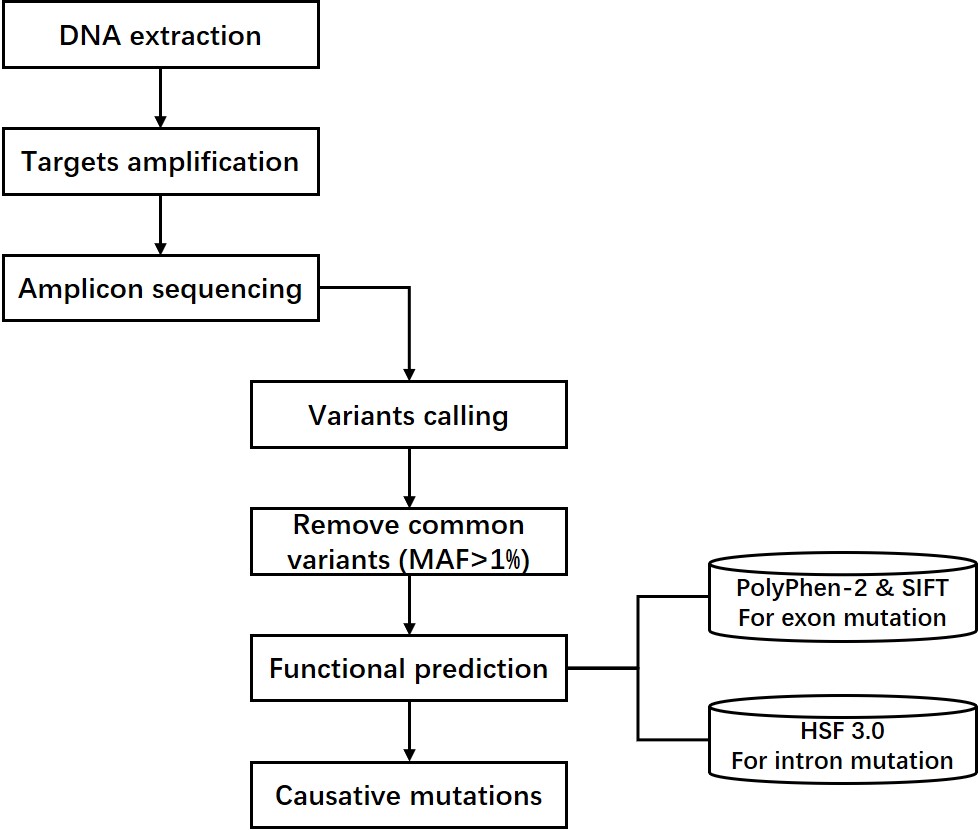

Supplement: Supplementary file 2 — Additional file 2: Figure S1. Workflow of detecting causative mutations for inherited disease. [file 12887_2019_1751_MOESM2_ESM.jpg]

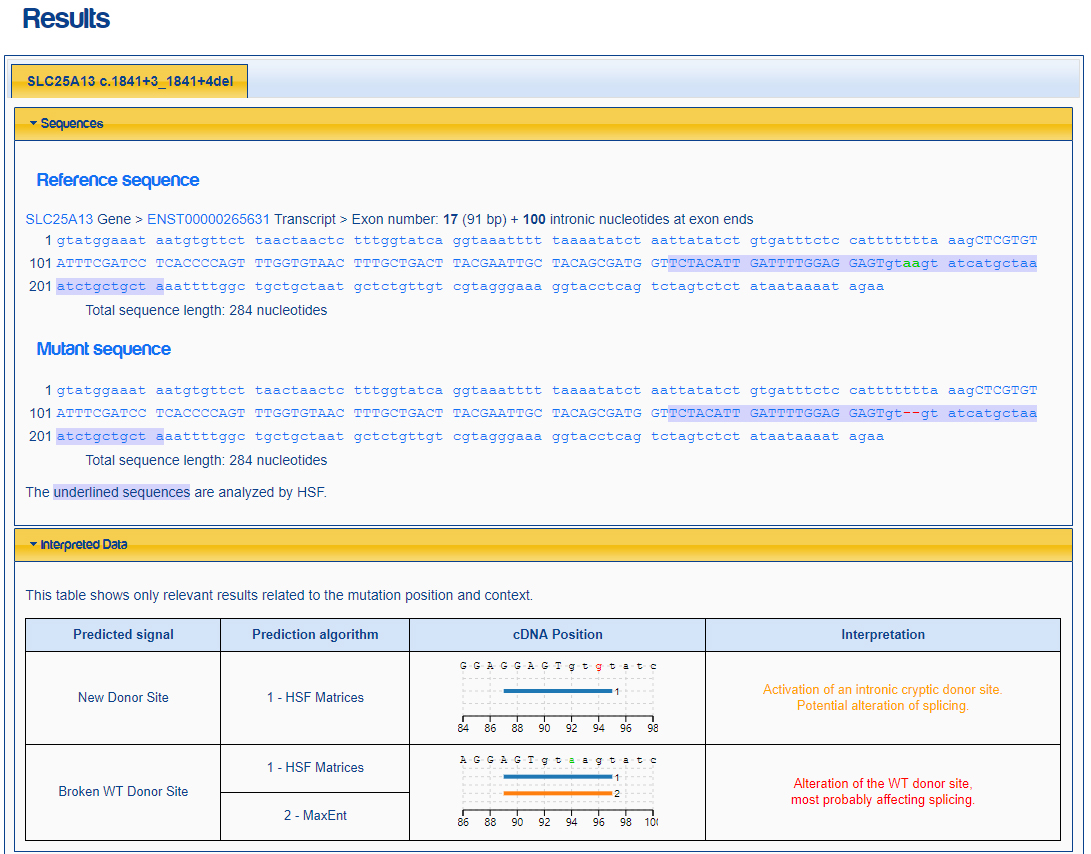

Supplement: Supplementary file 4 — Additional file 4: Figure S2. Result of functional prediction for SLC25A13:c.1841+3_1841+4delAA by using HSF. [file 12887_2019_1751_MOESM4_ESM.jpg]
