# Supplementary material for: Identification of a novel splicing mutation in the SLC25A13 gene from a patient with NICCD: a case report
Source: BMC Pediatr. 2019 Oct 13;19:348. doi: 10.1186/s12887-019-1751-9 (PMC6790242; doi:10.1186/s12887-019-1751-9)
Supplement: Supplementary file 3 — Additional file 3: Table S2. Result of splice-site prediction. [file 12887_2019_1751_MOESM3_ESM.docx]

Table S2. Result of splice-site prediction.

|  | HSF Matrices | MaxEnt |
| --- | --- | --- |
| Splice-site type | Donor | |
| Reference Motif | AGTgtaagt | |
| Mutate Motif | AGTgtgtat | |
| Reference Score | 79.79 | 8.46 |
| Mutate Score | 47.45 | -9.89 |
| Variation Ration | -40.53% | -216.9% |
| Interpretation | WT site broken | WT site broken |
